# Gender-based Alzheimer's detection using ResNet-50 and binary dragonfly algorithm on neuroimaging

**DOI:** 10.3389/frai.2025.1717913

**Published:** 2025-12-08

**Authors:** Muhammad Ikram ul Haq, Waqas Haider Bangyal, Arfan Jaffar, Asma Abdullah Alfayez, Adnan Ashraf, Meshari Alazmi, Mubbashar Hussain

**Affiliations:** 1 Department of Software Engineering, Superior University, Lahore, Pakistan; 2 Department of Information Technology, University of Gujrat, Gujrat, Pakistan; 3 Department of Computer Science, Kohsar University Murree, Murree, Pakistan; 4 King Abdullah International Medical Research Center, Riyadh, Saudi Arabia; 5 King Saud Bin Abdulaziz University for Health Science, Riyadh, Saudi Arabia; 6 Ministry of National Guard Health Affairs, Riyadh, Saudi Arabia; 7 School of Computer Science and Technology, Beijing Institute of Technology, Beijing, Haidian, China; 8 College of Computer Science and Engineering, University of Ha'il, Ha'il, Saudi Arabia

**Keywords:** Alzheimer's disease, fMRI, ADNI, pretrained, gender

## Abstract

Alzheimer's disease (AD) is an incurable, progressive neurodegenerative disorder. It is characterized by a gradual decline in memory, cognition, and behavior, which ultimately results in severe dementia and functional dependence. AD begins to develop in the brain at an early stage, while its symptoms appear gradually over time. Early diagnosis and classification of Alzheimer's is a critical research focus due to its silent progression. The current literature highlights a gap in gender-based studies, revealing that the risk of AD varies by gender, age, race, and ethnicity. The nature of the association between AD and these factors requires further exploration to better understand their impact on disease risk and progression. Effectively employing multiple algorithms is essential for accurate diagnosis of Alzheimer's development. This study proposed the GRDN model, which explored a critical aspect of gender-based Alzheimer's detection. To detect subtle changes in the brain, functional magnetic resonance imaging (fMRI) scans have been acquired from the ADNI dataset. In order to balance class distribution and enhance classifier performance on underrepresented groups, a generative adversarial network (GAN) is applied. A balanced dataset is provided to the ResNet-50 architecture for feature extraction, resulting in feature matrices set with a range of 100, 250, and 450. These feature set matrices were then fed to a swarm intelligence-based approach, the binary dragonfly algorithm (BDA), for feature selection, which identified the most informative features. After feature engineering, the resultant matrices of feature selection were provided to the five machine learning (ML) classification algorithms for data classification. The results show that as the size of the features set increases and the accuracy of the classification improves. The simulation results demonstrated that the fineKNN achieved strong performance, with an accuracy of 94.8% on the male group on a feature set of 450, and consistently outperformed other models across all study groups.

## 1 Introduction

Alzheimer's disease (AD) is a widespread neurodegenerative disorder affecting the lives of more than 55 million people across the globe, and its prevalence is projected to triple by 2050 (Chen Y. et al., 2024). Individuals with AD face a gradual loss of mental ability, including impaired memory, reduced reasoning, language disorder, and neuropsychiatric disorders such as apathy and anxiety. The most significant risk factor that affects individuals with AD is age, with risk increasing from age 50 onwards (Calderón-Garcidueñas, 2022).

Current research depicts that AD starts with minor alterations in the brain anatomy. One of the key characteristics of AD is the generation of abnormal proteins, mainly β-amyloid plaques and tau tangles, in a region of the brain called the hippocampus. Through the progression of this disease, these protein clumps spread to adjacent brain regions, causing shrinkage of the brain tissues, breaking down connections between cells, and leading to memory fade/loss problems (Rao et al., 2024). It is essential to note that these changes can typically be detected in the later stages of the disease, although this is not particularly helpful for early-stage diagnosis. Different neuroimaging modalities, such as Computed Tomography (CT) scans, Positron Emission Tomography (PET) scans, Magnetic Resonance Imaging (MRI), and Functional Magnetic Resonance Imaging (fMRI), are commonly used for AD detection. MRI provides clear structural images of brain tissues, revealing shrinkage and volume loss in the temporal lobe, which are key indicators of neurodegenerative disease. fMRI captures detailed functional information about the brain by measuring changes in blood flow and oxygen level, revealing which regions of the brain are active. fMRI utilizes blood oxygen level-dependent (BOLD) contrast to capture changes in oxygenation, distinguishing between oxygen-rich and oxygen-poor blood. PET scans work by visualizing and measuring metabolic activities in the brain by detecting radioactive signals emitted by radioactive tracers injected into the body. PET demonstrates how the body's cells are performing on a biochemical level, providing insight into metabolic processes. CT scans use X-ray methodology to create cross-sectional images of the brain (van Dyck et al., 2023; Chen Z. et al., 2024).

Researchers employed several publicly available datasets to further the scientific research, including Alzheimer's Disease Neuroimaging Initiative (ADNI), Open Access Series of Imaging Studies (OASIS), and Australian Imaging, Biomarker & Lifestyle Study (AIBL). The ADNI is the most used dataset that provides multimodal neuroimaging, biomarker, genetic, and clinical data of multiple modalities (Grigas et al., 2024). In order to categorize the different stages of AD, including cognitive normal (CN), mild cognitive impairment (MCI), and Alzheimer's disease (AD), ADNI provides a comprehensive neuroimaging and clinical dataset. Four different variants of the ADNI dataset progress with the timeline: ADNI-1 (2004–2009), ADNI-GO (2009–2011), an extension of ADNI-1, ADNI-2 (2011–2016), ADNI-3 (2016–2022), and ADNI-4 (2022–present) (Goyal et al., 2024).

This study uses fMRI scans from the ADNI-4 dataset, including novel remote cohorts that integrate screening and monitoring participants outside of a clinical setting. ADNI-4 includes three diagnostic groups—MCI, CN, and AD—comprising both brain images and clinical details. The dataset tracks how biomarkers change over time and examines factors such as age, gender, education, race, genetics, and marital status to understand their relationship to early signs of Alzheimer's. Collectively, all of these research elements aim to detect the disease at an earlier stage by making more accurate predictions and can better understand how Alzheimer's begins and progresses, with the goal of including a diversified population. The inclusion of remote cohorts provides greater opportunities to study how genotype influences AD risk across gender, age, race, and ethnicity (Beckett et al., 2024). This approach deepens the understanding of how this disease develops and progresses, and it supports tailored interventions for diverse groups.

**Figure 1:**
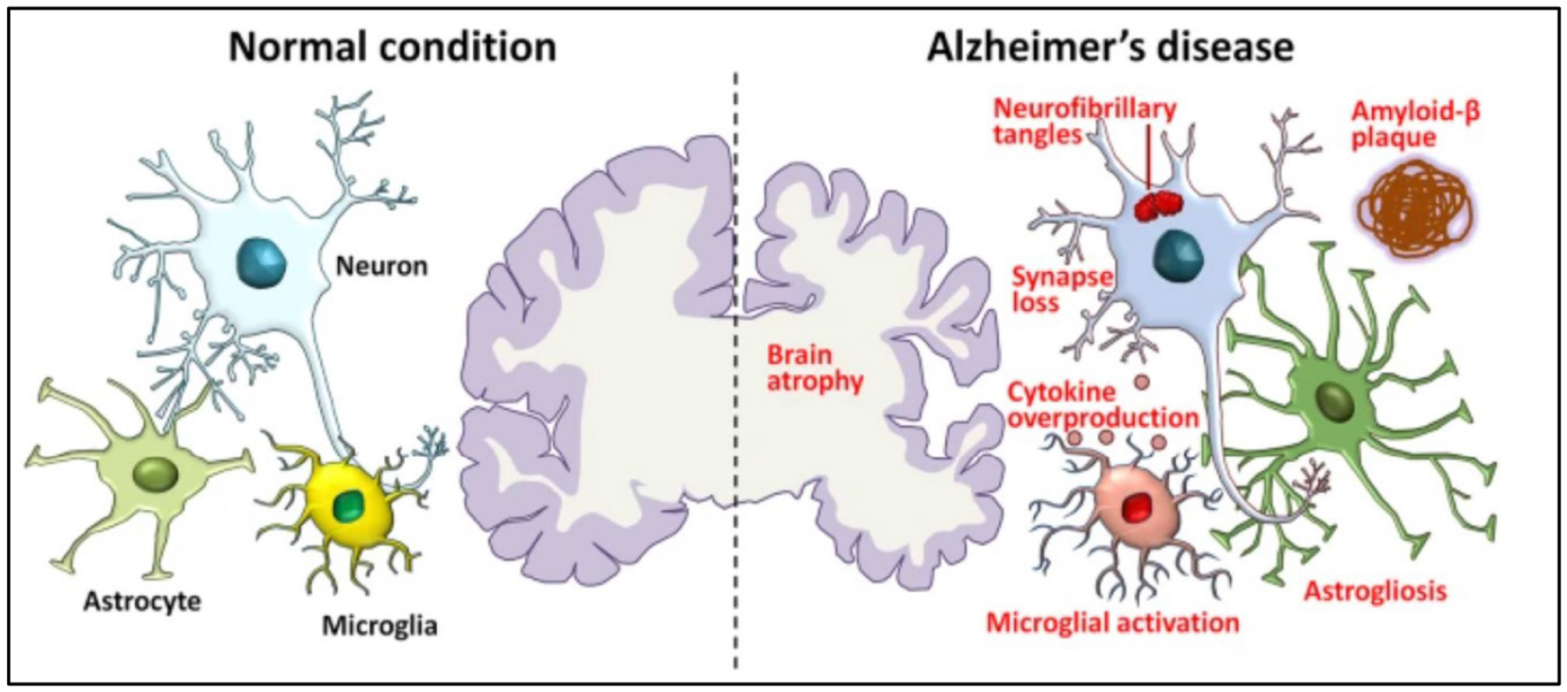
Pathological hallmarks of Alzheimer's disease, including brain atrophy, amyloid-β deposits, tau tangles, and synaptic loss (Xia et al., 2021).

When AD develops, the brain begins to undergo a progressive process called atrophy, characterized by the loss of neurons and the shrinking of brain tissue. Pathological changes in an AD brain and a normal brain are shown in Figure 1. In AD, amyloid-β builds up outside the neurons and neurofibrillary tangles inside due to the tau protein. As a result, synapses are lost and communication between neurons becomes weakened. This synaptic loss contributes to the cognitive decline seen in Alzheimer's patients.

Machine learning (ML) has emerged as one of the best approaches in health informatics, especially for the detection of Alzheimer's. There are many famous ML algorithms, such as Support Vector Machine (SVM), Decision Tree (DT), and Artificial Neural Networks (ANN), which are used to detect different factors such as age, gender, biomarkers, education, marital status, and genetic profiles for the detection of AD at an early stage (Jiang et al., 2024; Mishra et al., 2024). Alongside, deep learning (DL) has produced remarkably better results for Alzheimer's diagnosis. To find the complicated pattern in neuroimaging data, there exist robust architectures such as CNNs, VGG, ResNet, and MobileNet, which have shown a strong ability to classify AD (Ftoutou et al., 2023; Mohsen, 2025).

Incorporating chronological changes in biomarkers along with diverse remote cohorts in ADNI-4 enhances biomarker reliability and participant diversity (Dubois et al., 2023). The binary classification of AD remains unsuccessful in capturing the disease's severity and progression. Therefore, multi-classification is necessary to capture temporal changes in biomarkers and improve predictive accuracy. Abuhantash et al. (2025) emphasized that many ML models achieve comparatively good accuracy while classifying AD multi-stages, but predicting the true severity and progression remains challenging due to often ignored clinical and demographic factors such as gender, age, education, race, genotype, and marital status, which are crucial for personalization. Existing studies incorporated imbalanced datasets, leading to biased classification results, especially for underrepresented stages. Additionally, there is a lack of comparison between established and new datasets that include additional features affecting model performance (Danter, 2024).

Artificial intelligence relies on a massive amount of data to learn generalized patterns and make precise predictions on unknown data instances. In the medical domain, datasets are usually smaller and often contain class imbalance across different classes of disease (Alayba et al., 2024). These limitations reduce the accuracy of classification models. To address these problems, various data augmentation techniques are used to expand the dataset and improve model performance. Traditional techniques include rotation, scaling, flipping, and noise addition, while deep learning-based methods, such as GANs and variational autoencoders (VAEs), generate realistic synthetic images (Siddhartha, 2020).

A significant gap that has been identified in Schwartz and Weintraub (2021) is the absence of gender-specific analysis, which limits the understanding of how men and women may respond differently to AD treatments. It has also identified the limited use of biomarkers to distinguish AD, which further reduced diagnostic accuracy. Addressing these shortcomings emphasized the need for more robust approaches to improve the reliability of predictive models for AD research. Some studies show uneven performance of recent DL models with respect to gender and ethnicity for neurodegenerative disease. In AD classification, these studies show inconsistent and heterogeneous results, indicating that there are several factors that have not been fully explored (Hammonds et al., 2024). The aforementioned discussion highlights the key research gaps and outlines the following objectives to further this research study.

- To identify an effective multi-stage classification model for early diagnosis of Alzheimer's disease.
- To find an effective gender-based Alzheimer's disease prediction model.
- To compare the results of the proposed model with state-of-the-art approaches.

The research article is distributed in the following sections, where related work is discussed in Section 2, while research methodology is elaborated in Section 3. Results and discussions are presented in Section 4, while the conclusion and future directions are covered in Section 5.

## 2 Related work

Previously, multiple studies had been carried out for the detection of Alzheimer's based on various factors like age, gender, and biomarkers. For better understanding, potential research has been collected and discussed in the following section.

Different DL and ML algorithms typically require huge data to train models effectively, to achieve accurate results. A 3D CNN model was trained on a comparatively large dataset, ABIDE, in order to address the challenge of limited AD-specific fMRI data. Furthermore, this model was fine-tuned on a small ADNI subset, which achieved an accuracy of 76.56%. This output was 30% higher than without TL. It shows the effectiveness of the proposed model on limited AD data (Warren and Moustafa, 2025).

Bolla et al. (2023) compared multiple ML algorithms for detecting MCI. The rs-fMRI scans from a regional Hungarian cohort and ADNI are utilized by the researchers. They had studied three different brain activity measures from these scans: intrinsic connectivity to evaluate how brain areas connect and communicate, local correlation to evaluate how nearby brain areas show similar activity, and fractional amplitude of low-frequency fluctuations to assess the strength of natural low-speed brain signals. Furthermore, five feature selection algorithms were utilized to select important features from these three measures for their analysis. SVM achieved an accuracy of 87% on the local dataset while attaining 90% accuracy on the ADNI dataset. Random forest, on the other hand, produced more consistent results by producing 84% and 82% accuracy on the local and ADNI datasets, respectively.

Feature selection plays a crucial role in managing the high-dimensional and complex data linked with AD. The BDA acts as an efficient feature selection strategy, inspired by dragonfly swarming dynamics to identify the most informative biomarkers. By optimizing feature subsets, BDA improves classification accuracy, minimizes redundancy, and enhances the performance and reliability of DL based AD diagnosis models (Zhang et al., 2021).

This has become increasingly evident that a combination of different classifiers with multiple feature extractors produces improved performance. In a study, conversion from MCI to AD by combining sMRI and rs-fMRI neuroimages from the ADNI repository is analyzed. The researchers compared different ML methods for analyzing data based on graph theory with different classifiers, including random forest, KNN, and AdaBoost, to evaluate which classifier performs better. The integration of SVM with graph theory achieved the best accuracies: 84.71% for MCIc (converted) vs. MCInc (non-converted) and 89.8% from MCIc vs. AD (Sun et al., 2022).

The researchers utilized the benefits of advancement in ML methods in studying the early diagnosis of AD and to predict if MCI will progress to AD or remain stable. Zhou et al. (2024) adopted fMRI images from the ADNI dataset and integrated them with a brain atlas, Automated Anatomical Labeling (AAL), to construct functional connectivity. GAN had been employed to create more training data by generating new, realistic images. To analyze temporal changes in data, researchers used multi-layer long short-term memory (LSTM), a variant of NN that is best fit for temporal space analyses. The model achieved an accuracy of 93.5% for the classification of AD patients apart from healthy people, and 75.5% for distinguishing sMCI (stable) from pMCI (progressed).

In Chattopadhyay et al. (2024), the researchers evaluated whether combining genetic data with fMRI scans could improve the results. Multiple DL models used in earlier researches are explored. The rs-fMRI scans from the ADNI dataset were utilized for this research. The goal was to test whether integrating genetic data could enhance the predictive accuracy of the model. The evaluation, however, demonstrated that the inclusion of genetic data did not show any improvement. The model achieved an accuracy of 92.8%, indicating that the genetic information did not contribute to better performance compared to using imaging features alone.

ResNet-50 has become a crucial DL model for AD classification due to its powerful residual learning and deep feature extraction capabilities. Its ability to capture subtle and fine-grained structural brain changes enhances diagnostic accuracy and reliability. When integrated with advanced frameworks, ResNet-50 significantly improves accuracy across AD, MCI, and CN classification tasks, which demonstrates its clinical potential for early diagnosis, disease monitoring, and personalized treatment in AD research (Zhou et al., 2025).

The researchers aimed to predict amyloid positivity (Aβ+) in AD, MCI, and CN (Turrisi et al., 2024). They compared several ML models, including logistic regression, XGBoost, and a shallow artificial neural network. They also evaluated several DL approaches, such as 2D CNNs, 3D CNNs, CNN-based transfer learning, and 3D vision transformer, to evaluate their effectiveness in AD prediction. The study utilized two neuroimaging modalities, MRI and PET, from the ADNI repository. A total of 1847 subjects were included. The result depicted that the DL models trained on MRI data achieved promising performance with a balanced accuracy of 77.11%.

ResNet-50 has emerged as a widely adopted learning architecture in AD classification. In a study (Han, 2025), ResNet-50 has been effectively integrated with vision transformers to enhance both local and global feature representations from brain MRI scans. The research shows remarkable accuracy in classifying AD stages, underscoring ResNet-50 significance in improving early diagnosis and aiding clinical decision-making.

In this study, a new hybrid model called 3D-CNN-VSwinFormer was created to detect AD at an early stage. This model used a 3D Convolutional Neural Network with an attention module to focus on important brain features. MRI scans from the ADNI dataset are utilized to test this model. The proposed model demonstrated remarkable performance, achieving an accuracy of 92.92% and an area under the curve of 0.9660 in distinguishing AD patients from CN individuals (Wang et al., 2023).

To make the diagnosis better, the study (Zamani et al., 2022) tested five different 3D-CNN models that vary in the number of convolution layers they have. The researchers also used three types of augmentation: zooming, shifting, and rotating to create more training data to improve the model performance. The researcher utilized MRI scans from the ADNI dataset. The model with 8 convolution layers achieved an accuracy of 87.21%, showing excellent performance of the proposed model.

Toga et al. (2024) utilized a multimodal Mamba classifier to fuse PET scans with MRI by using a Pixel-Level Bi-Cross Attention mechanism to make a prediction. To overcome the high cost and limited access to PET scans, the researchers synthesized PET scans from MRI. An accuracy of 90.78% of AD prediction is achieved in this study by utilizing two datasets, OASIS and ADNI.

Deep neural networks are top-notch at separating Alzheimer's from healthy brain MRIs, but their complex design makes them hard to interpret. To fix that, Rivera Mindt et al. (2024) compared three heatmap methods: Layer-wise Relevance Propagation, Integrated Gradients, and Guided Grad-CAM by measuring how well each overlapped with a trusted meta-analysis map. All three outperformed traditional SVM-based brain maps, and Integrated Gradients showed the closest match.

Selecting the most effective parameters is a major challenge in neuroimaging research. This study proposes an optimization method using five evolutionary algorithms. To measure functional connectivity, rs-fMRI scans from the ADNI dataset are used in this study. Functional connectivity was analyzed using graph measures, resulting in 1,155 parameters. The evolutionary algorithms selected a subset of parameters for classification. A two-layer ANN was used for classification into healthy control and early mild cognitive impairment. The model achieved an accuracy of 94.55%, which shows the strength of rs-fMRI and evolutionary optimization methods (Toga et al., 2024).

Collectively, these recent studies from 2022 onward illustrate a dynamic and rapidly evolving landscape of ML techniques applied to ADNI fMRI data, leveraging hybrid DL, transfer learning, evolutionary optimization, multimodal fusion, sophisticated feature selection, and novel architectures such as transformers. These approaches significantly push forward the early and accurate detection of Alzheimer's disease and its prodromal stages, offering promising routes toward clinical translation.

## 3 Methodology

The most common dataset repositories available for the detection of Alzheimer's are ADNI, Kaggle, ABIDE, and OASIS. The ADNI study was initiated in 2004, and its multiple variants of the dataset are available (Turrisi et al., 2024). The different variants of the ADNI dataset are based on differences in the group of people and updates in the data collected over time. These changes occur due to new scientific findings and improvements in technology.

### 3.1 Dataset

**Figure 2:**
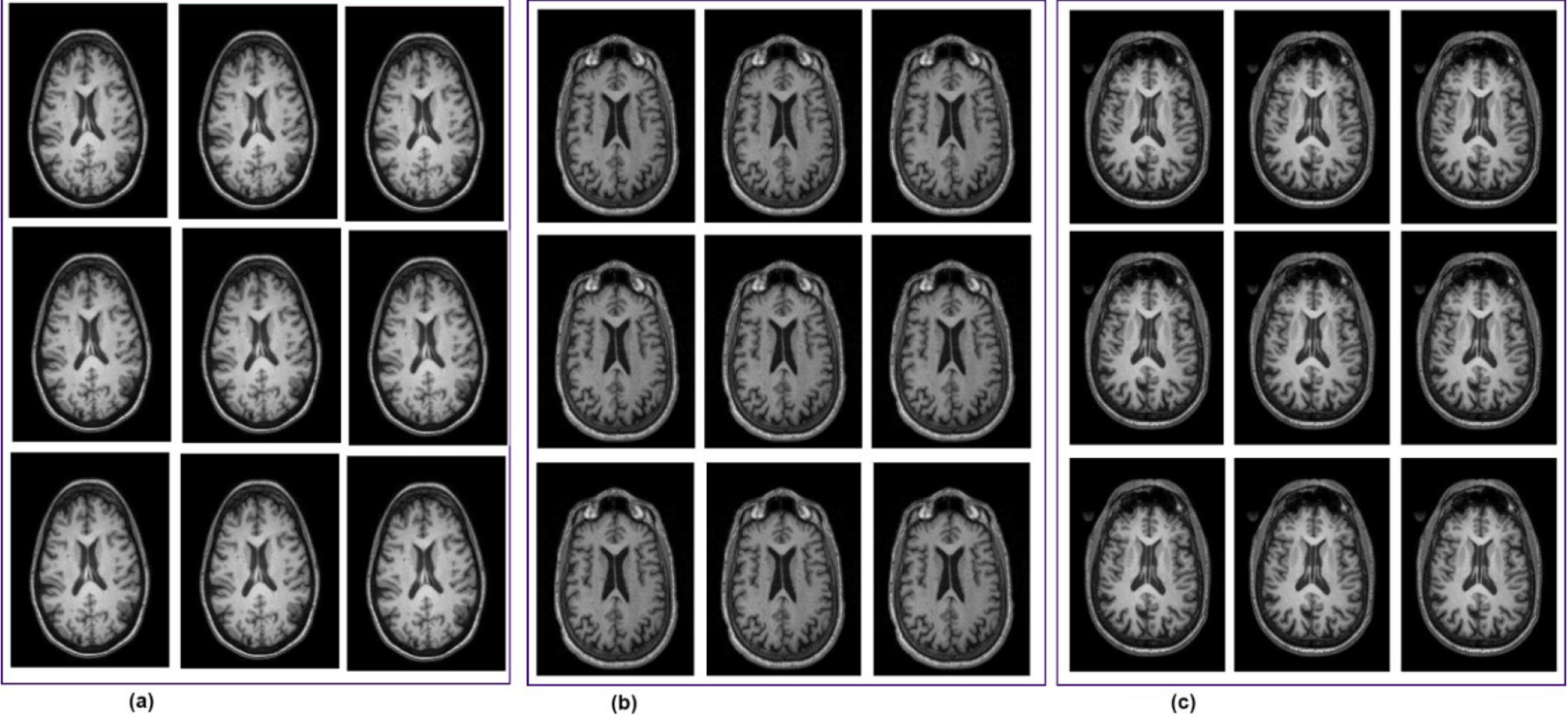
fMRI scans from the ADNI-4 dataset. **(a)** Cognitive normal. **(b)** Mild cognitive impairment. **(c)** Alzheimer's disease (AD).

This study utilized the Alzheimer's Disease Neuroimaging Initiative (ADNI) dataset, which by mid-2024 had contributed to over 6,000 peer-reviewed studies worldwide. Over its two decades of operation, ADNI has created more than 47,000 user accounts, with about 26,000 of them currently active, which reflects its global research and ongoing scientific relevance (Alatrany et al., 2024). The ADNI has progressed through several phases to better understand how Alzheimer's disease develops, using brain imaging and data from various groups of people. The first phase, ADNI-1 (2004–2009), gathered baseline data from people with Alzheimer's, healthy individuals, and those with MCI, using scans like DTI, MRI, and PET. ADNI-GO (2009–2011) built on this by focusing on people with early MCI and added functional MRI (fMRI) scans. In ADNI-2 (2011–2016), the study expanded to include more groups, such as late MCI and people with subjective memory complaints, while continuing to collect different types of brain scans to better track Alzheimer's progression. ADNI-3 (2016–2022) kept the same participant groups and scans but improved image quality by using more advanced 3T MRI machines. The latest phase, ADNI-4 (2022 to now), aims to include the underrepresented population through remote cohorts and digital follow-ups. It continues to collect detailed imaging data, enabling researchers to gain deeper insight into Alzheimer's disease across different populations. The ADNI-4 dataset contains three classes: MCI, CN, and AD (Sánchez et al., 2025; Borawar and Kaur, 2023). Representative samples of fMRI scans from the ADNI-4 dataset are represented in Figure 2.

**Figure 3:**
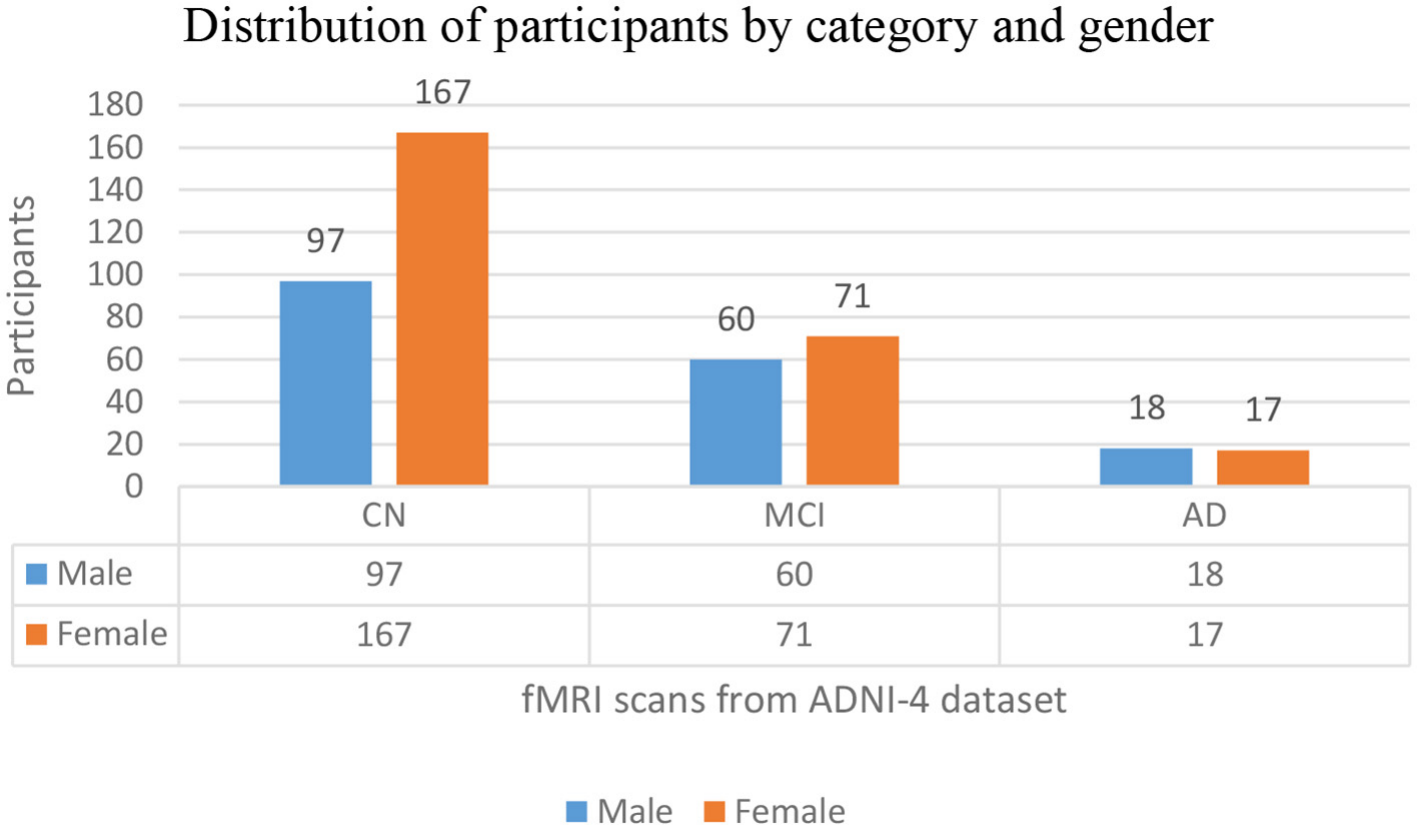
Graphical distribution of fMRI scans from the ADNI-4 dataset.

The distribution of participants in this study by their diagnostic categories and gender depicts that the total population of this study is 430, downloaded from https://ida.loni.usc.edu on 21 March 2025, where 175 are male participants (40%) and 255 are female participants (59%). There are 264 CN participants, 97 are male participants (22% of the total sample), and 167 are female participants (38%), making up 61% of all participants. Among the 131 MCI, 60 are male participants (13%) and 71 are female participants (16%), representing 30% of the total population. The AD group has 35 participants, with 18 male subjects (4%) and female subjects (3%), accounting for 8% of all subjects. In the dataset, female participants constitute a larger portion of the sample across all three categories. Among these categories, CN is the biggest group. Figure 3 depicts the graphical distribution of fMRI scans from the ADNI-4 dataset. An overview of the sample composition used in this research is shown in Table 1.

**Table 1:** Distribution of categories and gender.

**Category**	**Gender**		**Total**
	**Male**	**Female**	
CN [*n* (%)]	97 (22%)	167 (38%)	264 (61%)
MCI [*n* (%)]	60 (13%)	71(16%)	131 (30%)
AD [*n* (%)]	18 (4%)	17 (3%)	35 (8%)
Total [*n* (%)]	175 (40%)	255 (59%)	430 (100%)

### 3.2 Data balancing

Dataset size and class imbalance remain significant challenges, hindering model generalizability and the development of personalized treatment plans. These limitations highlight the need for better model design and richer data integration to improve prediction performance in AD (Raeisi et al., 2025; Wang, 2025). So, limited dataset size and class imbalance while working with the ADNI-4 dataset were the core challenges. To address both challenges, a deep learning-based augmentation technique, DCGAN, to generate novel fMRI scans has been implemented. It was conducted to address gender-based stratification with the ResNet-50 training process. Initially, the dataset was imbalanced between genders within each class. After applying DCGAN-based augmentation, AD [50.0% male individuals, 50% female individuals, CN (49.9% male individuals, 50.1% female individuals), and MCI (50.0% male individuals, 50.0% female individuals)] achieved balanced gender distribution, ensuring fair representation and reducing bias in ResNet-50 model training. The dataset originally contained 35 AD, 264 CN, and 250 AD. Utilizing DCGAN, 229 AD, 96 CN, and 119 MCI scans were generated, yielding a balanced dataset. The dataset was split at the subject level to avoid overlap between sets, with 70% of subjects used for training, 15% for validation, and 15% for testing. All image slices from the same subject were kept in the same set. The generator learned the feature distribution of training data through transposed convolutional layers and expanded them into latent vectors into high-dimensional volumes. During training, the model demonstrates significant convergence, effectively capturing the characteristics of fMRI while maintaining anatomical plausibility in generated images. The parameter settings of the GCGAN and their corresponding values are provided in Table 2.

**Table 2:** Hyperparameter settings for DCGAN.

**SR #**	**Hyperparameter**	**Value**
1	Batch size	64
2	Learning rate	0.0002
3	Optimizer	Adam, SoftMax
4	Number of epochs	100
5	Loss function	Cross-entropy
6	Normalization	Batch normalization
7	Data split	Subject level 70% trin 15% validation 15% test

### 3.3 Proposed DRGN model

**Figure 4:**
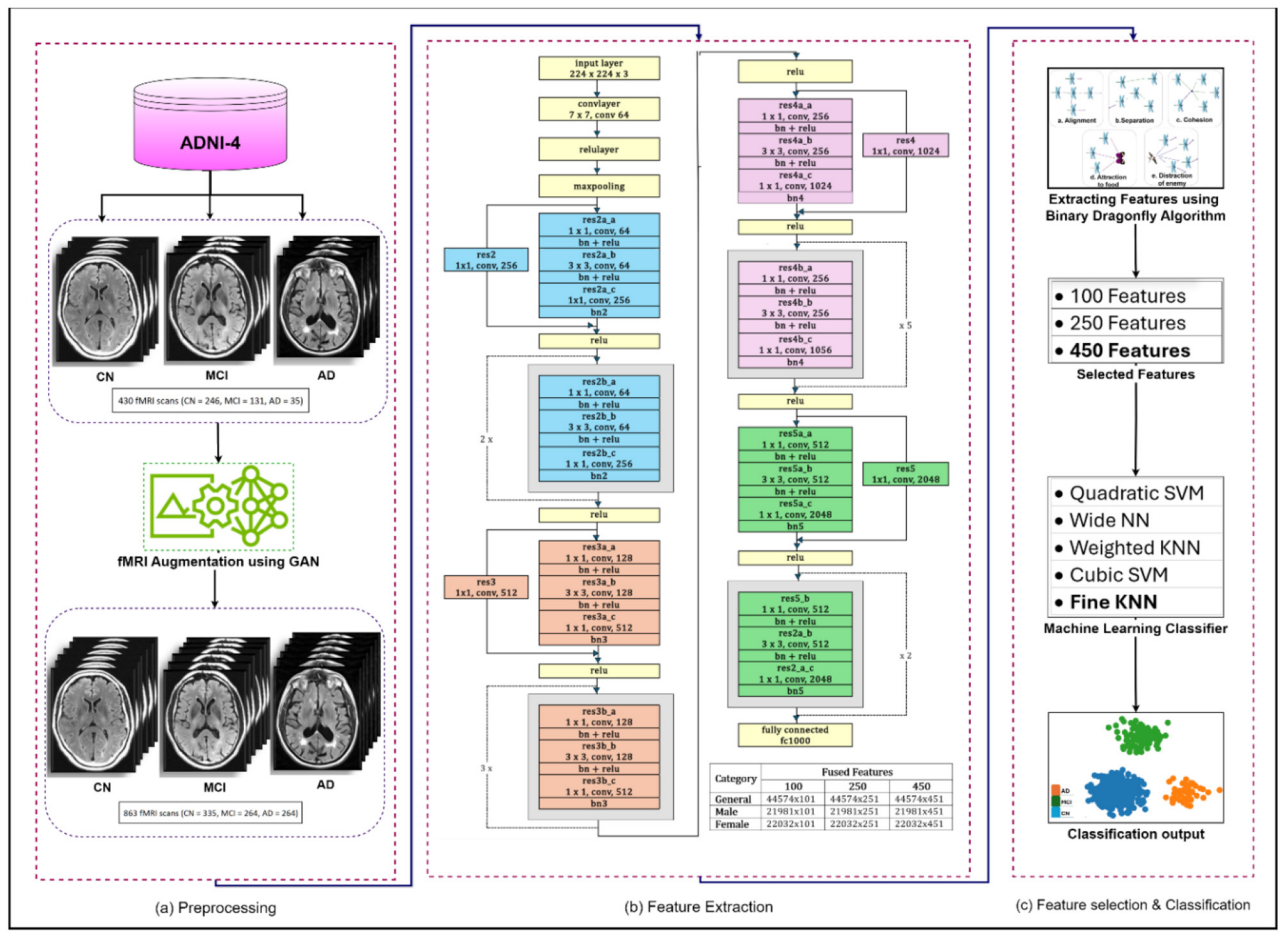
Proposed methodology for the detection of Alzheimer's disease. **(a)** Preprocessing. **(b)** Feature extraction. **(c)** Feature selection & classification.

The framework consists of four successive phases; preprocessing involves a comprehensive evaluation of fMRI sans form ADNI-4 dataset and the generation of synthetic images using an augmentation framework. Feature extraction is performed using ResNet-50, followed by BDA, generating feature sets of 100, 250, and 450. Finally, five ML classifiers were applied to ensure systematic evaluation and progression. The preprocessing phase involves removing non-brain tissue, commonly referred to as skull stripping, correcting head motion artifacts, standardizing anatomical alignment, and applying spatial smoothing to produce uniform, high-quality input data for analysis. As the fMRI scans in the ADNI-4 dataset exhibit class imbalance, a GAN was employed to generate additional synthetic fMRI data for the underrepresented class. The GAN develops a balance among classes of AD and reduces predication bias (Alshinwan et al., 2021). The diminution process protects important neurological patterns for categorization while eliminating lazy data. The proposed model uses multiple ML classifiers to process features extracted by ResNet-50 and features selected by BDA. The model established an affiliation between AD, MCI, and CN. ML algorithms are very well-suited for handling high-dimensional data distribution, such that found in fMRI neuroimaging scans. The ability of ML algorithms to process complex data to find patterns and relationships that might be difficult to distinguish otherwise. The proposed methodology of AD detection is illustrated in Figure 4.

**ResNet-50**: A 50-layer deep convolutional neural network architecture called ResNet-50 was developed by Microsoft Research in 2015. One of the key features of ResNet-50 is a residual connection, which is its ability to effectively train very deep networks by addressing the vanishing gradient problem (Cui et al., 2020). The residual architecture has been empirically proven to significantly improve generalization and feature representation, resulting in improved diagnostic accuracy and robustness in neuroimaging. The architecture consists of an initial convolutional layer followed by four residual blocks. Each block has a bottleneck design with three convolutional layers: a 1 × 1 convolution for dimensionality reduction, a 3 × 3 convolutional layer for spatial feature extraction, and another 1 × 1 convolutional layer to restore dimensions. After these, average pooling and a fully connected layer produce the final classification (Chantar et al., 2021). The capability of ResNet50 to handle spatial and temporal aspects of the data enhances the framework's performance in identifying AD-related changes in brain function (Ali et al., 2024).

The residual block, which is the main building unit of ResNet, simply adds the original input to the learned residual, making training easier. The mathematical expression of the residual block is shown in Equation 1, whereas after applying the activation function to the residual output, the next layer's input obtained is shown in Equation 2.


$$\begin{array}{l} y_{l}=h\left(x_{l}\right)+f\left(x_{l},W_{l}\right) \end{array}$$ (1)

$$\begin{array}{l} x_{l}+1=f\left(y_{l}\right) \end{array}$$ (2)

where *xl* denote the input feature, *Wl* weights and biases, *h*(*x*_*l*_) represents identity mapping, *f*(*x*_*l*_, *W*_*l*_) represents the residual function, and *f* is ReLU function.

By stacking multiple residual blocks, the final feature representation at the layer *l* is shown in Equation 3.


$$\begin{array}{l} x_{l}=x_{l}+\overset{L-1}{\underset{i=l}{\sum }}F\left(x_{i},w_{i}\right) \end{array}$$ (3)

where *x*_*l*_ indicates a series of matrix-vector products, and *L* represents the summation of the output of residual functions.

In ResNet, the connections allow gradients to flow directly during backpropagation. This prevents them from vanishing and makes training deep networks more effective. The feature *x*_*l*_ can be described as a series of matrix-vector products, where *L* represents the summation of the outputs of all preceding residual functions. This means that rather than learning an unreferenced function, each residual block learns a residual function to refine the input features collectively. The loss equation functions as E from the chain rule of backpropagation, as in Equation 4.


$$\begin{array}{l} \delta \frac{\delta E}{\delta xl\;}=\frac{\delta E}{\delta \;xL}\;\frac{\delta \;xL}{\delta xl}=\frac{E}{\delta \;xL}\;\left(1+\frac{\delta }{\delta xl}\;\overset{L-1}{\underset{i=l}{\sum }}\left(xi,\;wi\right)\right)\;\left(4\right) \end{array}$$ (4)

where $\frac{\delta E}{\delta xl}$ allows gradient to flow back without touching weights, $\frac{\delta E}{\delta xl\;}\left(\frac{\delta }{\delta xl}\;\overset{L-1}{\underset{i=l}{\sum }}\left(xi,\;wi\right)\right)$ progress through weight layers, and $\frac{\delta }{\delta xl}$ ensure the information is propagated back to any layer. Equation 4 also depicts that it is less likely for the gradient $\frac{\delta E}{\delta xl}\;$ to be canceled, it is so the term $\frac{\delta }{\delta xl}\;\overset{L-1}{\underset{i=l}{\sum }}\left(xi,\;wi\right)$, cannot constantly be equal to −1. It shows that the gradient does not vanish when the weights are small.

**Figure 5:**
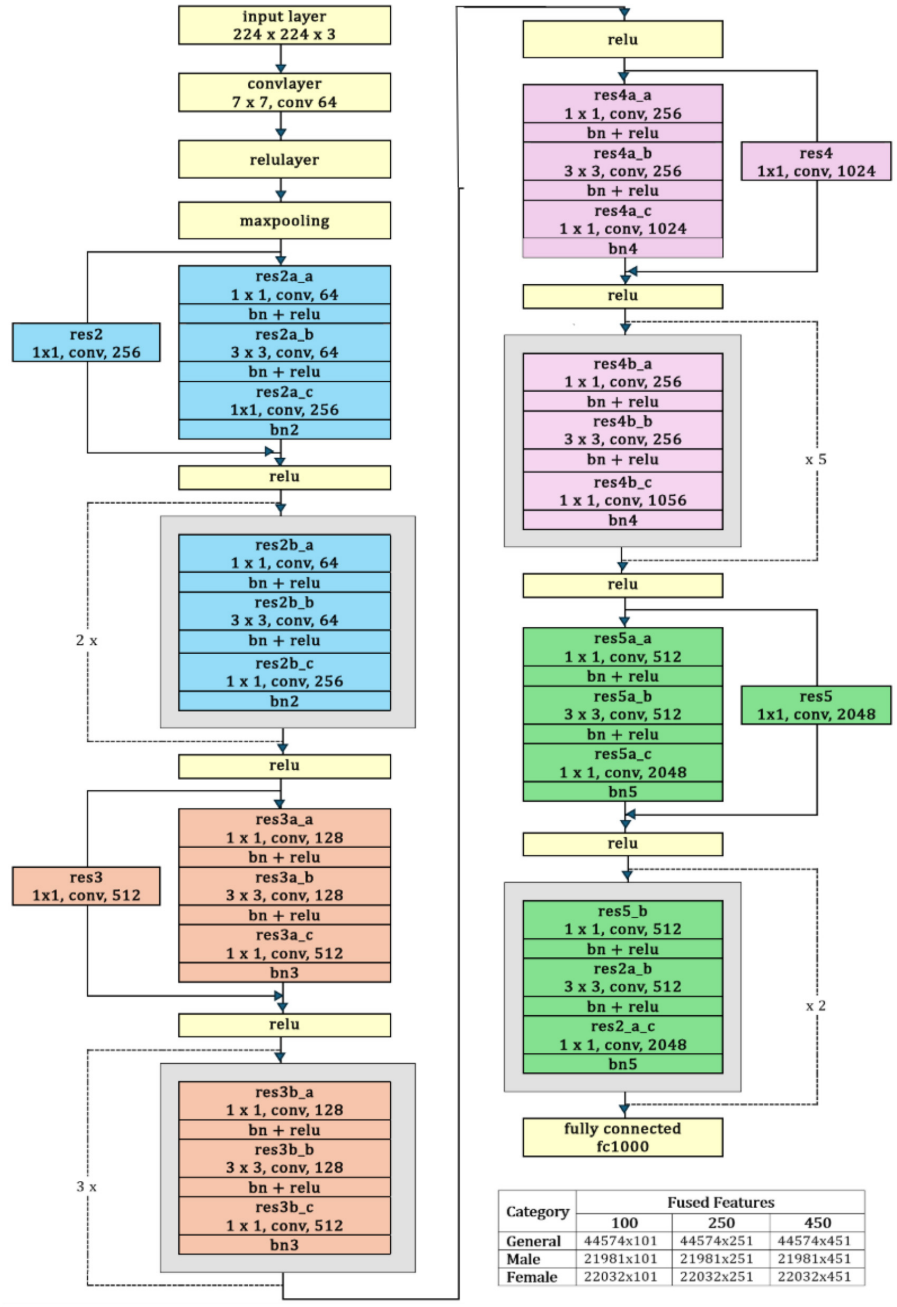
ResNet-50 architecture.

The ResNet-50 is used to extract deep discriminative features from ADNI-4 fMRI data, enhancing Alzheimer's classification accuracy. Figure 5 provides a schematic representation of the ResNet-50 architecture. Its hierarchical convolutional layers capture complex spatial patterns linked to disease progression. The model was trained using stochastic gradient descent with momentum (SGDM) optimizer with a learning rate of 0.01, batch size of 128, momentum of 0.9, L2 regularization of 1 × 10^−4^, and 30 epochs. These parameters ensured stable convergence and improved classification performance across AD, MCI, and CN groups. To help the model adapt to fMRI data, balance learning speed, enhance feature extraction, and improve generalization, the parameter setting and their values are summarized in Table 3.

**Table 3:** Hyperparameter settings for ResNet-50.

**Sr#**	**Hyperparameter**	**Value**
1	Optimizer	SGDM
2	Initial learning rate	0.01
3	Momentum	0.9
4	L2 regularization	1 × 10^−4^
5	Batch size	128
6	Epochs	30
7	Shuffle	Once per epoch

**Binary Dragonfly Algorithm**: A nature-inspired optimizer, Binary Dragonfly Algorithm (BDA) is a variant of the dragonfly algorithm, a swarm optimization technique introduced by Mirjalili in 2015. BDA addresses multiple optimization problems, including discrete, single-objective, and multi-objective problems (Sadiq et al., 2021). It is a nature-inspired algorithm based on the swarming behavior of dragonflies. The key concept is that each dragonfly represents a possible solution, which is based upon its movement, which is guided by behaviors like alignment, separation, cohesion, attraction to food, and avoidance of enemies. Each solution is evaluated through a fitness function, e.g., classification accuracy in feature selection (Bhutta et al., 2024).

Alignment describes associating the velocity of an individual dragonfly with respect to its neighboring dragonflies. The mathematical equation of alignment is written as Equation 5.


$$\begin{array}{l} A_{i}=\;\frac{\;\sum N\;j=1\;Vj}{N} \end{array}$$ (5)

where the velocity vector is referred to as *V*_*j*_.

Separation refers to the way dragonflies avoid collision. Mathematically, it can be written as in Equation 6.


$$\begin{array}{l} Si=-\overset{N}{\underset{j=1}{\sum }}\;X-X_{j} \end{array}$$ (6)

where *X* denotes the current search agent, while *X*_*j*_ refers to j-the neighbor of *X*. *N* is the number of neighbors.

Cohesion is sticking together with the average position of its neighbors. Mathematically, it is referred to as in Equation 7.


$$\begin{array}{l} C_{i}=\;\frac{\;\overset{N}{\underset{j=1}{\sum }}X_{j}}{N}-X \end{array}$$ (7)

Attraction to food is a natural pull toward the direction of the food location. This drift in the swarm toward food is shown in Equation 8.


$$\begin{array}{l} F_{i}=F_{location\;}-X \end{array}$$ (8)

where *F*_*location*_is the food source location, while *X* is the current member.

Distraction from the enemy is the desire of the dragonfly to flee from the enemy. Mathematically, it is defined as in Equation 9.


$$\begin{array}{l} E_{i}=E_{location\;}+X. \end{array}$$ (9)

In this equation *E*_*location*_is the position of the enemy, and *X* is the position of the current member (Zhu et al., 2022).

Several variants of ML classifiers, including Quadratic SVM, Cubic SVM, Wide NN, Weighted KNN, and Fine KNN, have been employed for neuroimaging classification tasks, as shown in Table 4.

**Table 4:** Variants of classifiers employed in different AD detection studies.

**Classifier**	**Variant**
SVM	Quadratic SVM
	Cubic SVM
NN	Wide NN
KNN	Weighted KNN
	Fine KNN

**Fine K-Nearest Neighbor** (Fine KNN) is an optimized variant of KNN designed to get improved predictive accuracy. This model incorporates fine-tuned hyperparameters, particularly the number of neighbors (*k)* and fine distance weighting strategies. In this study, Fine-KNN is applied to the selected feature set to distinguish between different classes of AD, providing a strong baseline for comparison.

**Weighed KNN** is a refined variant of the conventional KNN algorithm, designed to enhance classification performance by considering greater importance of nearby neighbors. This weighting strategy reduces the influence of noise and outliers. The studies depict that weighted KNN effectively captures variations in the brain, which are critical in distinguishing between AD, MCI, and CN when utilizing fMRI scans.

**Wide-Neural Network (**Wide-NN) employs a large number of hidden units in fewer layers, enabling the model to learn diverse feature interactions without deep stacking. In the case of fMRI images, this architecture is well-suited, as wide representations can capture complex spatial variation in brain activity. Moreover, it helps reduce overfitting and ensures better generalization across different stages of AD.

**Cubic SCM**: it uses a cubic kernel to map input data into higher dimensions, which makes it effective for handling complex, non-linear patterns. Cubic SVM can capture complicated relationships in neuroimaging features, making it well-suited for separating overlapping different classes where linear separation is insufficient.

**Quadratic SVM:** it offers a balance between linear and highly non-linear classifiers by applying a polynomial kernel of degree two. It works effectively for moderately complex decision boundaries, making it useful for distinguishing among various classes.

## 4 Results and discussion

In this study, a robust framework containing DCGAN + ResNet-50 + BDA + SVM evaluated information from the 430 preprocessed fMRI scans distributed across ADNI-4 global sites. While conventional 3D-CNN models demonstrated strong performance, the proposed GRDN model achieved an overall accuracy of 88.77%. When evaluated by gender, the model attained accuracies of 94.8% for male individuals and 93.0% for female individuals. By combining ResNet-50 with BDA and SVM, the model effectively extracted efficient spatiotemporal features and established a robust decision boundary, mitigating the typical challenges of analyzing neuroimaging data independently. Performance metrics such as precision, F1-score, recall, specificity, and accuracy confirmed the reliability of this proposed framework. Furthermore, residual learning and skip connections resolved the vanishing gradient problem, which means neural networks can now be trained much deeper. This helps the networks to learn and understand the data in a better and more detailed way, improving overall performance. By passing information directly from earlier layers to later, these techniques help the network keep important signals intact during training. This makes learning easier, improves overall performance, and allows the network to capture the data more efficiently. The framework faces challenges because brain scans are not always uniform. This difference depends on the hospital, the scanner model, or the chosen sequence settings. These inconsistencies in acquisition methods, scanner settings, resolution, and quality made it harder to build a framework that generalizes well across neuroimages. Additionally, the types and numbers of patient samples vary among various classes. These differences made it hard for the system to perform consistently and accurately every time because it struggled to adjust and work well with all the different types of data.

### 4.1 Experimental setup

The experimental setup comprises 430 fMRI scans and is conducted on a system running Windows11 Enterprise Edition, equipped with a Ryzen7 7700 processor, 32 GB RAM, 1 TB NVMe, and an NVIDIA RTX-3070ti GPU. MATLAB R2024a, a full-featured programming environment, was used for simulation and experimentation. This study employed ResNet50 for feature extraction, BDA for feature selection, and 5 classification algorithms, namely SVM, KNN, and NN, were used to predict AD. The extracted features for the general group, male group, and female group consisted of 100, 250, and 450 features, and are presented in Table 5. Architectural details of ResNet50 and parameters of BDA, used during the optimization process, are shown in Tables 6, 7, respectively. Among five classification algorithms, the highest accuracy of 94.8% was obtained using the Fine KNN classifier, as shown in **Table 10**.

**Table 5:** Extracted features using ResNet-50.

**Category**	**Fused features**		
	**100**	**250**	**450**
General	44,574 × 101	44,574 × 251	44,574 × 451
Male	21,981 × 101	21,981 × 251	21,981 × 451
Female	22,032 × 101	22,032 × 251	22,032 × 451

**Table 6:** Architectural details of ResNet-50.

**Layer name**	**Layer type**	**Feature map**	**Stride**	**Padding**	**Pooling**	**Learnables**
input_1	ImageInputLayer	[224 × 224 × 3]				—
conv1	Convolution2DLayer	[112 × 112 × 64]	[2 × 2]	[3 × 3 × 3 × 3]		Weights: [7 × 7 × 3 × 64]; Bias: [1 × 1 × 64]
bn_conv1	BatchNormalizationLayer	[112 × 112 × 64]				Scale: [1 × 1 × 64]; Offset: [1 × 1 × 64]
activation_1_relu	ReLULayer	[112 × 112 × 64]				—
max_pooling2d_1	MaxPooling2DLayer	[56 × 56 × 64]	[2 × 2]	[1 × 1 × 1 × 1]	Ma × Pooling [3 × 3]	—
res2a_branch2a	Convolution2DLayer	[56 × 56 × 64]	[1 × 1]	[0 × 0 × 0 × 0]		Weights: [1 × 1 × 64 × 64]; Bias: [1 × 1 × 64]
bn2a_branch2a	BatchNormalizationLayer	[56 × 56 × 64]				Scale: [1 × 1 × 64]; Offset: [1 × 1 × 64]
activation_2_relu	ReLULayer	[56 × 56 × 64]				—
res2a_branch2b	Convolution2DLayer	[56 × 56 × 64]	[1 × 1]	[1 × 1 × 1 × 1]		Weights: [3 × 3 × 64 × 64]; Bias: [1 × 1 × 64]
bn2a_branch2b	BatchNormalizationLayer	[56 × 56 × 64]				Scale: [1 × 1 × 64]; Offset: [1 × 1 × 64]
activation_3_relu	ReLULayer	[56 × 56 × 64]				—
res2a_branch2c	Convolution2DLayer	[56 × 56 × 256]	[1 × 1]	[0 × 0 × 0 × 0]		Weights: [1 × 1 × 64 × 256]; Bias: [1 × 1 × 256]
res2a_branch1	Convolution2DLayer	[56 × 56 × 256]	[1 × 1]	[0 × 0 × 0 × 0]		Weights: [1 × 1 × 64 × 256]; Bias: [1 × 1 × 256]
bn2a_branch2c	BatchNormalizationLayer	[56 × 56 × 256]				Scale: [1 × 1 × 256]; Offset: [1 × 1 × 256]
bn2a_branch1	BatchNormalizationLayer	[56 × 56 × 256]				Scale: [1 × 1 × 256]; Offset: [1 × 1 × 256]
activation_4_relu	ReLULayer	[56 × 56 × 256]				—
res2b_branch2a	Convolution2DLayer	[56 × 56 × 64]	[1 × 1]	[0 × 0 × 0 × 0]		Weights: [1 × 1 × 256 × 64]; Bias: [1 × 1 × 64]
bn2b_branch2a	BatchNormalizationLayer	[56 × 56 × 64]				Scale: [1 × 1 × 64]; Offset: [1 × 1 × 64]
activation_5_relu	ReLULayer	[56 × 56 × 64]				—
res2b_branch2b	Convolution2DLayer	[56 × 56 × 64]	[1 × 1]	[1 × 1 × 1 × 1]		Weights: [3 × 3 × 64 × 64]; Bias: [1 × 1 × 64]
bn2b_branch2b	BatchNormalizationLayer	[56 × 56 × 64]				Scale: [1 × 1 × 64]; Offset: [1 × 1 × 64]
activation_6_relu	ReLULayer	[56 × 56 × 64]				—
res2b_branch2c	Convolution2DLayer	[56 × 56 × 256]	[1 × 1]	[0 × 0 × 0 × 0]		Weights: [1 × 1 × 64 × 256]; Bias: [1 × 1 × 256]
bn2b_branch2c	BatchNormalizationLayer	[56 × 56 × 256]				Scale: [1 × 1 × 256]; Offset: [1 × 1 × 256]
activation_10_relu	ReLULayer	[56 × 56 × 256]				—
res3a_branch2a	Convolution2DLayer	[28 × 28 × 128]	[2 × 2]	[0 × 0 × 0 × 0]		Weights: [1 × 1 × 256 × 128]; Bias: [1 × 1 × 128]
bn3a_branch2a	BatchNormalizationLayer	[28 × 28 × 128]				Scale: [1 × 1 × 128]; Offset: [1 × 1 × 128]
activation_11_relu	ReLULayer	[28 × 28 × 128]				—
res3a_branch2b	Convolution2DLayer	[28 × 28 × 128]	[1 × 1]	[1 × 1 × 1 × 1]		Weights: [3 × 3 × 128 × 128]; Bias: [1 × 1 × 128]
bn3a_branch2b	BatchNormalizationLayer	[28 × 28 × 128]				Scale: [1 × 1 × 128]; Offset: [1 × 1 × 128]
activation_12_relu	ReLULayer	[28 × 28 × 128]				—
res3a_branch2c	Convolution2DLayer	[28 × 28 × 512]	[1 × 1]	[0 × 0 × 0 × 0]		Weights: [1 × 1 × 128 × 512]; Bias: [1 × 1 × 512]
res3a_branch1	Convolution2DLayer	[28 × 28 × 512]	[2 × 2]	[0 × 0 × 0 × 0]		Weights: [1 × 1 × 256 × 512]; Bias: [1 × 1 × 512]
bn3a_branch2c	BatchNormalizationLayer	[28 × 28 × 512]				Scale: [1 × 1 × 512]; Offset: [1 × 1 × 512]
bn3a_branch1	BatchNormalizationLayer	[28 × 28 × 512]				Scale: [1 × 1 × 512]; Offset: [1 × 1 × 512]
activation_13_relu	ReLULayer	[28 × 28 × 512]				—
res3b_branch2a	Convolution2DLayer	[28 × 28 × 128]	[1 × 1]	[0 × 0 × 0 × 0]		Weights: [1 × 1 × 512 × 128]; Bias: [1 × 1 × 128]
bn3b_branch2a	BatchNormalizationLayer	[28 × 28 × 128]				Scale: [1 × 1 × 128]; Offset: [1 × 1 × 128]
activation_14_relu	ReLULayer	[28 × 28 × 128]				—
res3b_branch2b	Convolution2DLayer	[28 × 28 × 128]	[1 × 1]	[1 × 1 × 1 × 1]		Weights: [3 × 3 × 128 × 128]; Bias: [1 × 1 × 128]
bn3b_branch2b	BatchNormalizationLayer	[28 × 28 × 128]				Scale: [1 × 1 × 128]; Offset: [1 × 1 × 128]
activation_15_relu	ReLULayer	[28 × 28 × 128]				—
res3b_branch2c	Convolution2DLayer	[28 × 28 × 512]	[1 × 1]	[0 × 0 × 0 × 0]		Weights: [1 × 1 × 128 × 512]; Bias: [1 × 1 × 512]
bn3b_branch2c	BatchNormalizationLayer	[28 × 28 × 512]				Scale: [1 × 1 × 512]; Offset: [1 × 1 × 512]
activation_16_relu	ReLULayer	[28 × 28 × 512]				—
activation_22_relu	ReLULayer	[28 × 28 × 512]				—
res4a_branch2a	Convolution2DLayer	[14 × 14 × 256]	[2 × 2]	[0 × 0 × 0 × 0]		Weights: [1 × 1 × 512 × 256]; Bias: [1 × 1 × 256]
bn4a_branch2a	BatchNormalizationLayer	[14 × 14 × 256]				Scale: [1 × 1 × 256]; Offset: [1 × 1 × 256]
activation_23_relu	ReLULayer	[14 × 14 × 256]				—
res4a_branch2b	Convolution2DLayer	[14 × 14 × 256]	[1 × 1]	[1 × 1 × 1 × 1]		Weights: [3 × 3 × 256 × 256]; Bias: [1 × 1 × 256]
bn4a_branch2b	BatchNormalizationLayer	[14 × 14 × 256]				Scale: [1 × 1 × 256]; Offset: [1 × 1 × 256]
activation_24_relu	ReLULayer	[14 × 14 × 256]				—
res4a_branch2c	Convolution2DLayer	[14 × 14 × 1,024]	[1 × 1]	[0 × 0 × 0 × 0]		Weights: [1 × 1 × 256 × 1,024]; Bias: [1 × 1 × 1,024]
res4a_branch1	Convolution2DLayer	[14 × 14 × 1,024]	[2 × 2]	[0 × 0 × 0 × 0]		Weights: [1 × 1 × 512 × 1,024]; Bias: [1 × 1 × 1,024]
bn4a_branch2c	BatchNormalizationLayer	[14 × 14 × 1,024]				Scale: [1 × 1 × 1,024]; Offset: [1 × 1 × 1,024]
bn4a_branch1	BatchNormalizationLayer	[14 × 14 × 1,024]				Scale: [1 × 1 × 1,024]; Offset: [1 × 1 × 1,024]
activation_25_relu	ReLULayer	[14 × 14 × 1,024]				—
res4b_branch2a	Convolution2DLayer	[14 × 14 × 256]	[1 × 1]	[0 × 0 × 0 × 0]		Weights: [1 × 1 × 1,024 × 256]; Bias: [1 × 1 × 256]
bn4b_branch2a	BatchNormalizationLayer	[14 × 14 × 256]				Scale: [1 × 1 × 256]; Offset: [1 × 1 × 256]
activation_26_relu	ReLULayer	[14 × 14 × 256]				—
res4b_branch2b	Convolution2DLayer	[14 × 14 × 256]	[1 × 1]	[1 × 1 × 1 × 1]		Weights: [3 × 3 × 256 × 256]; Bias: [1 × 1 × 256]
bn4b_branch2b	BatchNormalizationLayer	[14 × 14 × 256]				Scale: [1 × 1 × 256]; Offset: [1 × 1 × 256]
activation_27_relu	ReLULayer	[14 × 14 × 256]				—
res4b_branch2c	Convolution2DLayer	[14 × 14 × 1,024]	[1 × 1]	[0 × 0 × 0 × 0]		Weights: [1 × 1 × 256 × 1,024]; Bias: [1 × 1 × 1,024]
bn4b_branch2c	BatchNormalizationLayer	[14 × 14 × 1,024]				Scale: [1 × 1 × 1,024]; Offset: [1 × 1 × 1,024]
activation_40_relu	ReLULayer	[14 × 14 × 1,024]				—
res5a_branch2a	Convolution2DLayer	[7 × 7 × 512]	[2 × 2]	[0 × 0 × 0 × 0]		Weights: [1 × 1 × 1,024 × 512]; Bias: [1 × 1 × 512]
bn5a_branch2a	BatchNormalizationLayer	[7 × 7 × 512]				Scale: [1 × 1 × 512]; Offset: [1 × 1 × 512]
activation_41_relu	ReLULayer	[7 × 7 × 512]				—
res5a_branch2b	Convolution2DLayer	[7 × 7 × 512]	[1 × 1]	[1 × 1 × 1 × 1]		Weights: [3 × 3 × 512 × 512]; Bias: [1 × 1 × 512]
bn5a_branch2b	BatchNormalizationLayer	[7 × 7 × 512]				Scale: [1 × 1 × 512]; Offset: [1 × 1 × 512]
activation_42_relu	ReLULayer	[7 × 7 × 512]				—
res5a_branch2c	Convolution2DLayer	[7 × 7 × 2,048]	[1 × 1]	[0 × 0 × 0 × 0]		Weights: [1 × 1 × 512 × 2,048]; Bias: [1 × 1 × 2,048]
res5a_branch1	Convolution2DLayer	[7 × 7 × 2,048]	[2 × 2]	[0 × 0 × 0 × 0]		Weights: [1 × 1 × 1,024 × 2,048]; Bias: [1 × 1 × 2,048]
bn5a_branch2c	BatchNormalizationLayer	[7 × 7 × 2,048]				Scale: [1 × 1 × 2,048]; Offset: [1 × 1 × 2,048]
bn5a_branch1	BatchNormalizationLayer	[7 × 7 × 2,048]				Scale: [1 × 1 × 2,048]; Offset: [1 × 1 × 2,048]
activation_43_relu	ReLULayer	[7 × 7 × 2,048]				—
res5b_branch2a	Convolution2DLayer	[7 × 7 × 512]	[1 × 1]	[0 × 0 × 0 × 0]		Weights: [1 × 1 × 2,048 × 512]; Bias: [1 × 1 × 512]
bn5b_branch2a	BatchNormalizationLayer	[7 × 7 × 512]				Scale: [1 × 1 × 512]; Offset: [1 × 1 × 512]
activation_44_relu	ReLULayer	[7 × 7 × 512]				—
res5b_branch2b	Convolution2DLayer	[7 × 7 × 512]	[1 × 1]	[1 × 1 × 1 × 1]		Weights: [3 × 3 × 512 × 512]; Bias: [1 × 1 × 512]
bn5b_branch2b	BatchNormalizationLayer	[7 × 7 × 512]				Scale: [1 × 1 × 512]; Offset: [1 × 1 × 512]
activation_45_relu	ReLULayer	[7 × 7 × 512]				—
res5b_branch2c	Convolution2DLayer	[7 × 7 × 2,048]	[1 × 1]	[0 × 0 × 0 × 0]		Weights: [1 × 1 × 512 × 2,048]; Bias: [1 × 1 × 2,048]
bn5b_branch2c	BatchNormalizationLayer	[7 × 7 × 2,048]				Scale: [1 × 1 × 2,048]; Offset: [1 × 1 × 2,048]
fc1000	FullyConnectedLayer	[1 × 1 × 1,000]				Weights: [1,000 × 2,048]; Bias: [1,000 × 1]

**Table 7:** Parameters of the binary dragonfly algorithm (BDA) and their dynamic value ranges used during the optimization process.

**Parameter**	**Value/range**
w (Inertia weight)	Decreases linearly from 0.9 → 0.4
Rate	Decreases linearly from 0.1 → 0 (not below 0)
s (Separation weight)	Random in [0, 2 × rate]
a (Alignment weight)	Random in [0, 2 × rate]
c (Cohesion weight)	Random in [0, 2 × rate]
f (Food attraction weight)	Random in [0, 2]
e (Enemy distraction weight)	Equal to the rate

The BDA was tuned with a population size of 100 and 100 iterations, where the inertia weight (w) decreased from 0.9 to 0.4, the rate from 0.01 to 0, and behavioral weights were randomized as s, a, c ε [0, 2 × rate], f ε [0. 2], and e = rate. The stopping criterion was reaching the maximum iterations or observing no improvement in the fitness function. Parameters of BDA, used during the optimization process, are shown in Table 7.

### 4.2 Performance metrics

The proposed GRDN model accessed the ADNI-4 dataset using a confusion matrix, widely regarded as the benchmark for evaluation. A confusion matrix contains true positives or true negatives for correct classification and false positives and false negatives for incorrect classification. Key performance indicators used to assess the model are presented in Table 8.

**Table 8:** The key performance indicators (KPIs) used to assess the performance of the model.

**KPIs**	**Description**
Accuracy	(True Positives + True Negatives)/Total Scans
F1 Score	2^*^ (Precision^*^ Recall/Precision + Recall)
Precision	True Positives/(True Positives + False Positives)
Specificity	True Negatives/(True Negatives + False Positives)
Recall	True Positives/(True Positives + False Negatives)

### 4.3 Classification results

In this study, a comprehensive framework for AD classification, focusing on both early diagnosis and gender-specific prediction, is evaluated. The proposed approach integrates DCGAN to address class imbalance, ResNet-50 for feature extraction, and BDA for feature selection. Extracted features were then classified using five ML classifiers, and the performance of the proposed model is compared with the state-of-the-art methods to assess its effectiveness.

#### 4.3.1 General analysis of the ADNI-4 dataset

**Figure 6:**
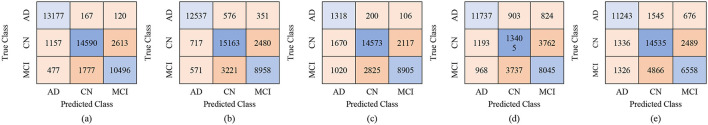
Confusion matrices using 100 selected features from the ADNI-4 dataset: **(a)** Fine KNN, **(b)** Cubic SVM, **(c)** Weighted KNN, **(d)** Wide NN, and **(e)** Quadratic SVM.

**Figure 7:**
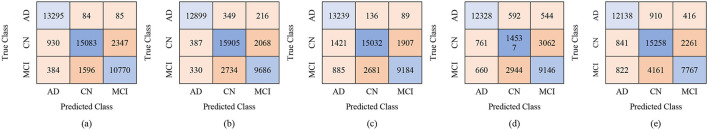
Confusion matrices using 250 selected features from the ADNI-4 dataset: **(a)** Fine KNN, **(b)** Cubic SVM, **(c)** Weighted KNN, **(d)** Wide NN, and **(e)** Quadratic SVM.

**Figure 8:**
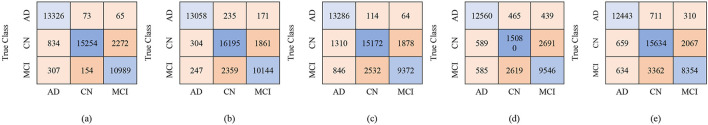
Confusion matrices using 450 selected features from the ADNI-4 dataset: **(a)** Fine KNN, **(b)** Cubic SVM, **(c)** Weighted KNN, **(d)** Wide NN, and **(e)** Quadratic SVM.

The proposed GRDN model uses ResNet-50 for feature extraction, BDA for feature selection, and then applies different machine learning classifiers for multi-class classification of AD, MCI, and CN. The results demonstrate how classifier choice impacts the general classification performance of these three classes. With 100 selected features, performance was moderate, but as the feature set increased to 250 and 450 features, both accuracy and robustness improved. Results in Table 9 show Fine KNN consistently outperformed others, reaching the highest accuracy of 88.8% (PER = 88.49, REC = 89.42, SPEC = 94.39) with 450 features. This shows not only a strong ability to detect true cases (high recall, fewer false negatives) but also an excellent ability to avoid mislabeling healthy subjects as diseased (high specificity, fewer false positives). Cubic SVM followed closely, achieving 88.4% accuracy with balanced precision and recall, meaning it maintained a stable trade-off between false positives and false negatives. On the other hand, Weighted KNN and the Wide Neural Network achieved accuracies of 84.9% and 83.4%, respectively, but their lower recall and specificity values indicate higher rates of both false positives (wrongly identifying CN as diseased) and false negatives (failing to detect AD or MCI). The Quadratic SVM performed the weakest (81.7% accuracy), producing the most frequent misclassifications. Overall, the analysis highlights that false positives are more harmful clinically because they may cause unnecessary anxiety or treatment for healthy subjects, while false negatives risk missing actual patients. Our results suggest that Fine KNN and Cubic SVM strike the best balance, with Fine KNN slightly ahead in accuracy and error control, making them more reliable for multi-class Alzheimer's classification. The confusion matrices generated using 100, 250, and 500 selected features are shown in Figures 6–8, respectively.

**Table 9:** Classification performance for Alzheimer's disease detection using ResNet-50 combined with BDA and different ML algorithms.

**Model**	**Classification performance using 100 selected features**					**Classification performance using 250 selected features**					**Classification performance using 450 selected features**				
	**PER**	**REC**	**F1**	**SPEC**	**ACC**	**PER**	**REC**	**F1**	**SPEC**	**ACC**	**PER**	**REC**	**F1**	**SPEC**	**ACC**
Quadratic SVM	72.57	71.37	71.49	85.68	72.54	79.12	78.06	78.3	88.97	78.9	82.06	81.03	81.31	90.44	81.7
Wide NN	74.14	74.43	74.28	86.98	74.45	80.61	80.82	80.71	90.20	80.8	83.26	83.43	83.34	91.54	83.4
Weighted KNN	81.95	82.31	81.81	90.94	82.2	83.84	84.08	83.69	91.86	84	84.67	84.94	84.56	92.29	84.9
Cubic SVM	82.21	81.99	82.05	90.83	82.24	86.47	86.13	86.27	92.92	86.4	88.49	88.25	88.35	93.98	88.4
**Fine KNN**	85.52	86.55	85.88	92.91	**85.50**	87.52	88.46	87.87	93.91	**87.80**	88.49	89.42	88.84	94.39	**88.80**

Bold values indicate the highest performance metrics.

#### 4.3.2 Gender-based analysis of ADNI-4 dataset

The dataset was further analyzed by dividing subjects into male and female groups to study gender-based classification performance.

#### 4.3.3 Male group

**Figure 9:**
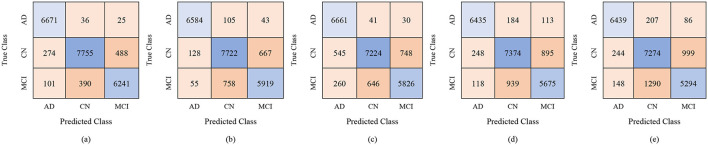
Confusion matrices for gender-wise classification (Male group) using 100 selected features from the ADNI-4 dataset: **(a)** Fine KNN, **(b)** Cubic SVM, **(c)** Weighted KNN, **(d)** Wide NN, and **(e)** Quadratic SVM.

**Figure 10:**
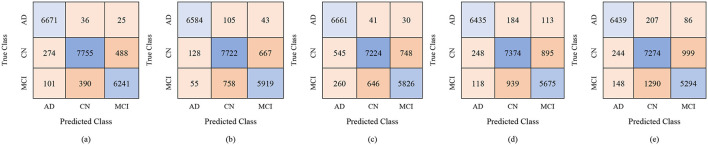
Confusion matrices for gender-wise classification (Male group) using 250 selected features from the ADNI-4 dataset: **(a)** Fine KNN, **(b)** Cubic SVM, **(c)** Weighted KNN, **(d)** Wide NN, and **(e)** Quadratic SVM.

**Figure 11:**
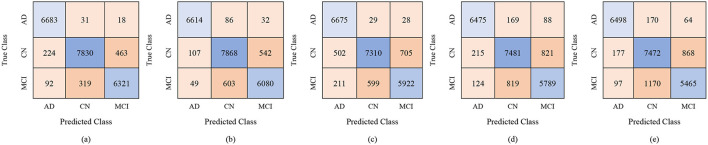
Confusion matrices for gender-wise classification (Male group) using 450 selected features from the ADNI-4 dataset: **(a)** Fine KNN, **(b)** Cubic SVM, **(c)** Weighted KNN, **(d)** Wide NN, and **(e)** Quadratic SVM.

For the male group, the multi-class classification results using different feature dimensions (100, 250, and 450 features) show consistent improvements as the number of selected features increases. Table 10 shows that, with 100 selected features, Fine KNN achieved the best performance with an accuracy of 92.4%, precision of 92.34%, and recall of 92.74%, closely followed by Cubic SVM with an accuracy of 89.1%. Weighted KNN, Wide Neural Network, and Quadratic SVM showed moderate performance, with accuracies ranging from 80.2% to 88.1%. When the feature dimension was increased to 250, the Cubic SVM model emerged as the top performer with 94% accuracy, 93.96% precision, and 94.28% recall, indicating its strong ability to balance false positives and false negatives. Fine KNN also remained competitive with 92% accuracy, while Wide NN and Weighted KNN achieved around 89–90% accuracy. Finally, with 450 selected features, the best performance was observed from Fine KNN, which reached an impressive 94.8% accuracy, 94.71% precision, and 95.03% recall, followed closely by Cubic SVM with 93.5% accuracy. Weighted KNN and Wide NN also performed well, crossing 89–90% accuracy, while Quadratic SVM achieved 88.4% accuracy. Figures 9–11 represent the confusion matrices obtained using 100, 250, and 500 selected features, respectively.

**Table 10:** Classification performance for Alzheimer's disease detection in the Male group using ResNet-50 combined with BDA and different ML algorithms.

**Model**	**Classification performance using 100 selected features**					**Classification performance using 250 selected features**					**Classification performance using 450 selected features**				
	**PER**	**REC**	**F1**	**SPEC**	**ACC**	**PER**	**REC**	**F1**	**SPEC**	**ACC**	**PER**	**REC**	**F1**	**SPEC**	**ACC**
Quadratic SVM	80.39	80.29	80.29	89.87	80.2	86.73	86.56	86.62	93.07	86.5	88.73	88.48	88.58	94.05	88.4
Wide NN	83.86	84.00	83.93	91.75	83.7	89.58	90.1	89.72	94.84	89.7	89.93	90.0	89.96	94.83	89.8
Weighted KNN	87.95	88.54	88.09	94.03	88.1	88.77	88.82	88.8	94.22	88.6	90.47	90.98	90.62	95.28	90.6
Cubic SVM	89.25	89.29	89.27	94.45	89.1	93.96	94.28	94.09	97.0	**94.0**	93.67	93.65	93.66	96.7	93.5
**Fine KNN**	92.34	92.74	92.5	96.2	**92.4**	92.18	92.13	92.15	95.91	92.0	94.71	95.03	94.85	97.39	**94.8**

Bold values indicate the highest performance metrics.

Overall, these results suggest that increasing the number of selected features leads to more robust and stable classification performance in the male group, with Fine KNN and Cubic SVM consistently outperforming other models. High precision and recall values indicate that these models not only detect true positives effectively but also minimize false positives, making them reliable for distinguishing among AD, MCI, and CN subjects in male participants.

##### 4.3.3.1 Female group

**Figure 12:**
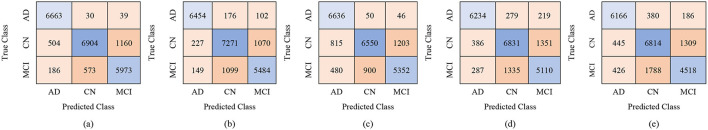
Confusion matrices for gender-wise classification (Female group) using 100 selected features from the ADNI-4 dataset: **(a)** Fine KNN, **(b)** Cubic SVM, **(c)** Weighted KNN, **(d)** Wide NN, and **(e)** Quadratic SVM.

**Figure 13:**
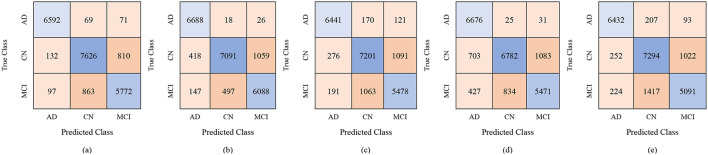
Confusion matrices for gender-wise classification (Female group) using 250 selected features from the ADNI-4 dataset: **(a)** Fine KNN, **(b)** Cubic SVM, **(c)** Weighted KNN, **(d)** Wide NN, and **(e)** Quadratic SVM.

**Figure 14:**
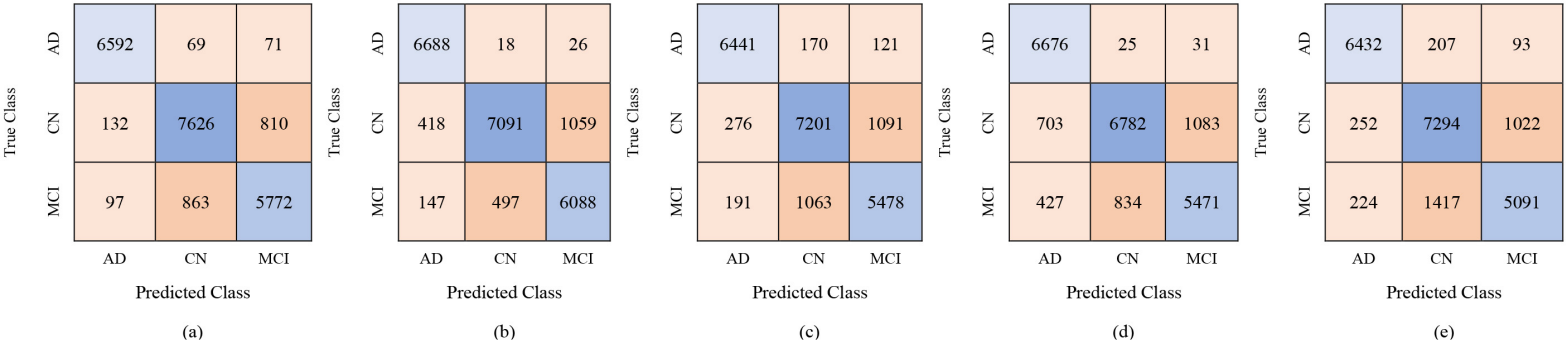
Confusion matrices for gender-wise classification (Female group) using 450 selected features from the ADNI-4 dataset: **(a)** Fine KNN, **(b)** Cubic SVM, **(c)** Weighted KNN, **(d)** Wide NN, and **(e)** Quadratic SVM.

The classification results for the female group, in a multi-class framework using the proposed model of ResNet50 for feature extraction, BDA for feature selection, and multiple ML classifiers show strong performance across different feature set sizes (100, 250, and 450). These results highlight how increasing the number of selected features improves the overall accuracy and robustness of the models for distinguishing AD, MCI, and CN. With 100 selected features, Fine KNN achieved the best performance, with an accuracy of 88.7%, precision of 88.62%, and recall of 89.43%, showing its reliability in female-based classification as shown in Table 11. Cubic SVM also performed strongly (ACC = 87.2%), while Weighted KNN and Wide NN had moderate results (ACC = 84.1% and 82.5%). Quadratic SVM showed the lowest accuracy (79.4%), reflecting challenges in handling limited feature dimensions. When the feature set was expanded to 250 features, the performance improved notably. Cubic SVM emerged as the top-performing model with an accuracy of 90.7%, precision of 90.84%, and recall of 90.89%, showing balanced generalization across classes. Fine KNN also performed comparably well (ACC = 90.2%), while Wide NN and Weighted KNN achieved reasonable accuracies (86.8% and 85.9%, respectively). Quadratic SVM followed closely with an accuracy of 85.4%. These results suggest that 250 selected features provide a richer and more discriminative representation for gender-based classification. With 450 features, the classification performance reached its peak. Cubic SVM achieved the highest accuracy of 92.9% along with strong precision (93.03%) and recall (93.06%). Fine KNN also performed very well with an accuracy of 91.5%, followed by Wide NN (ACC = 89.1%) and Quadratic SVM (ACC = 88.5%). Weighted KNN, while slightly lower, still achieved a solid 87.4% accuracy. These results confirm that larger feature sets enhance the discriminative ability of the proposed ResNet50-BDA pipeline in female subjects. In terms of false positives and false negatives, models with higher recall, such as Fine KNN and Cubic SVM, minimize false negatives, ensuring fewer AD or MCI cases are missed. High specificity, especially above 95% in Cubic SVM and Fine KNN (with 250–450 features), reduces false positives, preventing CN subjects from being misclassified as diseased. Overall, the results demonstrate that the proposed ResNet50-BDA framework performs robustly in gender-based classification. In the female group, the models achieved up to 92.9% accuracy, confirming the framework's effectiveness for distinguishing AD, MCI, and CN with different feature selection scales. Increasing the number of features significantly enhances classification performance, with Cubic SVM emerging as the most reliable classifier. The performance visualization through confusion matrices, based on 100, 250, and 500 selected features, is illustrated in Figures 12–14.

**Table 11:** Classification performance for Alzheimer's disease detection in the Female group using the proposed ResNet-50 combined with BDA and different ML algorithms.

**Model**	**Classification performance using 100 selected features**					**Classification performance using 250 selected features**					**Classification performance using 450 selected features**				
	**PER**	**REC**	**F1**	**SPEC**	**ACC**	**PER**	**REC**	**F1**	**SPEC**	**ACC**	**PER**	**REC**	**F1**	**SPEC**	**ACC**
Quadratic SVM	79.54	79.41	79.37	89.48	79.4	85.64	85.43	85.48	92.51	85.4	88.79	88.58	88.65	94.11	88.5
Weighted KNN	84.03	84.84	84.11	92.11	84.1	85.79	86.53	85.9	92.98	85.9	87.27	87.98	87.41	93.72	87.4
Wide NN	82.55	82.75	82.64	91.12	82.5	86.83	87.03	86.93	93.29	86.8	89.12	89.28	89.19	94.45	89.1
Fine KNN	88.62	89.43	88.81	94.39	**88.7**	90.1	90.85	90.3	95.13	90.2	91.41	92.08	91.6	95.78	91.5
**Cubic SVM**	87.32	87.4	87.36	93.47	87.2	90.84	90.89	90.86	95.27	**90.7**	93.03	93.06	93.05	96.39	**92.9**

Bold values indicate the highest performance metrics.

### 4.4 Comparison with state-of-the-art algorithm

Table 12 presents a comparative summary of recent ML classifiers applied to AD using neuroimaging data from prominent datasets such as ADNI, ABIDE, and OASIS. Notably, 3D-CNN has revealed strong performance, with accuracy ranging from 87.21% on MRI data to 92.92% on the ADNI MRI dataset and up to 92.80% when trained on fMRI data. ANN applied to MRI exhibited relatively lower accuracy of 77.10%, indicating variability in model effectiveness across architectures. More complex hybrid models such as the combination of GIG-ICA with 3D CNN attained accuracy of 76.56% on a combined ADNI and ABIDE fMRI dataset. This reflects challenges related to data heterogeneity and limited sample sizes. Multimodal approaches like the Mamba classifier integrating MRI and PET data from ADNI and OASIS reached an accuracy of 90.78%, highlighting the advantages of combining imaging modalities. The model trained on the male group (450 features) achieved superior performance and is evaluated against state-of-the-art models. The classification approach focusing on the male subgroup with rs-fMRI data from the ADNI-4 dataset, employing a ResNet-50 backbone combined with the BDA and fine-tuned K-Nearest Neighbors (KNN), achieving the highest accuracy of 94.80%. This shows that a gender-specific model with DL can accurately predict using fMRI neuroimaging.

**Table 12:** Comparison with state-of-the-art algorithm.

**References**	**Year**	**Classifier**	**Dataset**	**Modality**	**Accuracy (%)**
Warren and Moustafa (2025)	2025	3D CNN	ADNI + ABIDE	fMRI	76.56
Bolla et al. (2023)	2023	SVM	ADNI	fMRI	90
Sun et al. (2022)	2021	Graph theory + SVM	ADNI	MRI+ fMRI	89.8
Zhou et al. (2024)	2022	CNN + LSTM	ADNI	fMRI	93.5
Chattopadhyay et al. (2024)	2024	3D-CNN	ADNI	fMRI	92.8
Turrisi et al. (2024)	2024	ANN	ADNI	MRI + PET	77.1
Wang et al. (2023)	2025	3D-CNN	ADNI	MRI	92.92
Zamani et al. (2022)	2024	3D-CNN	ADNI	MRI	87.21
Toga et al. (2024)	2025	Mamba classifier	ADNI + OSAIS	MRI + PET	90.78
Rivera Mindt et al. (2024)	2023	CNN + SVM	ADNI	MRI	90.1
Toga et al. (2024)	2022	EA + ANN	ADNI	MRI+ fMRI	94.55
Proposed Model	2025	ResNet-50+ BDA+ Fine KNN	ADNI-4	rs-fMRI	**94.80**

Bold values indicate the highest performance metrics.

## 5 Conclusion

This study proposed a GRDN framework to address a critical research gap by exploring gender-based analysis in AD classification using fMRI data taken from the ADNI-4 dataset. Furthermore, the silent progression of AD by emphasizing accurate early diagnosis is examined, which is crucial for timely intervention and improved patient care. The dragonNet50 framework integrates ResNet-50 for deep feature extraction, the BDA for optimal feature selection, and multiple ML classifiers for final classification. The results demonstrated that this approach is effective in distinguishing between AD, MCI, and CN subjects. Subsequently, GAN to generate synthetic images, which helped balance the dataset and improve stability in classification performance, was employed. For general classification across the three classes AD, MCI, and CN, our framework showed strong accuracy, precision, recall, and F1-scores, highlighting its robustness and generalization ability. When analyzing gender-based classification, some differences were observed between female and male groups. For the male group, the FineKNN classifier achieved the best performance, with accuracy values above 94% when using 250 and 450 selected features. Cubic SVM and Weighted KNN also performed competitively, while Wide NN and Quadratic SVM showed moderate results. In the female group, the performance was slightly lower than the male group but still showed promising outcomes. For instance, Cubic SVM reached an accuracy of 92.9% with 450 selected features and 90.7% with 250 features. Weighted KNN and Wide NN performed reasonably well, though not at the level of Fine KNN and Cubic SVM. These findings suggest that gender-specific variations may play a role in classification accuracy and should be explored further in future studies. These results indicate that the model not only distinguishing between the classes effectively but also maintains stability across varying sample distributions. The strong performance across multiple metrics highlights the robustness, reliability, and generalization capability of the proposed framework in handling complex neuroimaging data. The results from this study suggested that our hybrid ResNet-50 + BDA + ML classifier framework provides an efficient and reliable approach for AD, MCI, and CN classification. The integration of GAN for synthetic data generation further strengthened the results by tackling the imbalance issue. The gender-based analysis revealed that male subjects showed slightly better classification accuracy compared to female subjects, indicating the importance of considering biological and demographic factors in neuroimaging studies.

Future research should focus on validating this framework on larger and multi-site datasets, incorporating longitudinal data to track disease progression, and applying explainable AI methods to enhance transparency and clinical trust. Moreover, to improve patient-specific diagnostic accuracy, future healthcare models should be trained on edge devices using federated learning, incorporating neuroimaging data, biomarkers, and region-specific demographic information. This approach ensures data privacy, minimize latency, and enables precise, personalized diagnostics tailored to individual patient profiles.

## Data Availability

Publicly available datasets were analyzed in this study. This data can be found at: https://adni.loni.usc.edu/data-samples/adni-data/(ADNI).
